# ﻿Terrestrial isopods (Isopoda, Oniscidea) of Slovakia: an annotated checklist, new records, and bibliography

**DOI:** 10.3897/zookeys.1225.125027

**Published:** 2025-02-05

**Authors:** Andrej Mock, Karel Tajovský, Adrián Purkart, Ivan Hadrián Tuf

**Affiliations:** 1 Department of Zoology, Faculty of Science, Institute of Biology and Ecology, Pavol Jozef Šafárik University, Šrobárova 2, SK-04154 Košice, Slovakia Pavol Jozef Šafárik University Košice Slovakia; 2 Biology Centre CAS, Institute of Soil Biology and Biogeochemistry, Na Sádkách 7, CZ-37005 České Budějovice, Czech Republic Biology Centre CAS, Institute of Soil Biology and Biogeochemistry České Budějovice Czech Republic; 3 Department of Zoology, Faculty of Natural Sciences, Comenius University, Mlynská dolina, Ilkovičova 6, SK-84215 Bratislava 4, Slovakia Comenius University Bratislava Slovakia; 4 Department of Ecology and Environmental Sciences, Faculty of Science, Palacký University Olomouc, Šlechtitelů 27, CZ-77900 Olomouc, Czech Republic Palacký University Olomouc Olomouc Czech Republic

**Keywords:** Carpathian-Pannonian region, chorotypes, distribution, ecological classification, list of species, woodlice

## Abstract

Woodlice (terrestrial isopods) represent an unmissable, often very numerous components of macrosaprophages inhabiting soil, rocky environments, rotting wood and subterranean habitats, as well as the dwellings of various organisms, including humans. The paper provides a comprehensive summary of the literature on woodlice in Slovakia, including a list of species with unpublished data on two exotic species first recorded in Slovakia. Research on Slovakian terrestrial isopods has been documented in 213 papers (from 1794 to 2024) and 25 theses. To date, 48 species from 14 families have been recorded in Slovakia. Of these, 30 species are autochthonous, while 18 species have been introduced into anthropogenic habitats, with eight species found exclusively in greenhouses. The list also includes an analysis of habitat preferences, bioindicator potential, and biogeographic comments. Notably, two species, *Buddelundiellacataractae* and *Reductoniscuscostulatus*, are recorded in Slovakia for the first time. Carpathian elements are sparsely distributed within the mosaic of well-preserved natural forests. *Hyloniscusmariae* is likely the only surface-dwelling endemic species in the northern parts of the Carpathian range. Other species, such as *Hyloniscustranssilvanicus*, *Trichoniscuscarpaticus* and *Trachelipusdifficilis*, have a broader distribution within the Carpathians. Additionally, *Mesoniscusgraniger*, *Orthometoponplanum* and a few other taxa reach their northern distribution limits in Slovakia. Current research is predominantly focused on the fauna of subterranean habitats and urbanized landscapes.

## ﻿Introduction

Slovakia (the Slovak Republic) is a small, landlocked country situated in Central Europe. Historically, it has been part of significant state entities, such as the Kingdom of Hungary and later the Austro-Hungarian Monarchy. In 1918, Slovakia joined with Czechia to form the Czechoslovak Republic. During World War II (1938–1945), the southern parts of Slovakia were annexed by the Kingdom of Hungary, while an independent Slovak Republic was established. After the war, Slovakia became part of Czechoslovakia until 1993, when it became an independent country.

This historical context is crucial when examining faunistic data. Faunistic publications from the region have been authored in various languages, including Hungarian, Slovak, German, Czech, Polish, and English. Historically, the rugged and less urbanized landscape attracted zoologists, including isopodologists. Researchers from neighbouring countries such as Hungary, Austria, Czechia, and Germany, with local authors, have contributed to the exploration of Slovak isopod fauna.

The country’s geography is diverse, with the flattened and mostly deforested landscape of the Pannonian (Danube) Lowland in the south bordered by the forested Carpathian Mountains in the north. The Carpathians are a significant extra-Mediterranean European biodiversity hotspot, often acting as a barrier for thermophilous species distribution and a crossroads for woodland faunas of different origins.

Rapid environmental changes, habitat destruction and the spread of allochthonous species (neobiota) underscore the importance of studying sensitive biota, such as soil arthropods like woodlice. Despite numerous references to terrestrial isopods in the literature, these soil arthropods have historically received inadequate attention. The lack of recent faunistic surveys, questionable historical data and ambiguities regarding species origins and endemism in Slovakia (covering the northern parts of the Carpathians and the northernmost tips of the Pannonian Lowland), with a paucity of ecological data on species, have resulted in an incomplete understanding of woodlouse fauna. During the past two decades, knowledge of isopods in Slovakia has significantly increased, but a comprehensive synthesis is still lacking. This gap in knowledge prompted the creation of this paper.

## ﻿History

There are only minimal records of terrestrial isopods from the territory of present-day Slovakia before the 20^th^ century. The first information on woodlice in the broader region of what is now Slovakia dates to the 18^th^ century, mentioning taxa from the genera “*Oniscus*” and “*Porcellio*” (Grossinger 1794: 353). Significant faunistic publications began appearing in the mid-19^th^ century. One of the earliest was by medical doctor Gustáv M. Reuss in 1853–1854, who documented the fauna of the town of Revúca in a large manuscript. This unpublished work was partly reviewed by Uhrin and Panigaj (2017), listing two common synanthropic species, *Porcellioscaber* (as *Porcellioasellus*) and *Oniscusasellus* (as *Oniscusmurarius*).

Cornelius Chyzer (1858), an expert on the natural history of present-day Eastern Slovakia, identified seven species in former Hungary, but only *Porcellioscaber* was localised to the town of Bardejov (Bartfeld) in northeast Slovakia (Chyzer 1858). Other sporadic data from the period came from the Tatra Mountains (Nowicki 1867) and regions bordering Poland (Dyduch 1903, 1904), as well as small towns such as Lučenec (= Losoncz; Malesevics 1892) and Banská Štiavnica (= Selmeczbánya; Petricskó 1892).

Little additional information was provided until the end of World War I and the Austro-Hungarian Monarchy. Dollfus (1901) reviewed terrestrial isopods in the National Museum in Budapest, documenting four common species from regions now in Slovakia. Ortvay (1902) reviewed the fauna of the former Pozsony (Pressburg or Bratislava) County, noting terrestrial isopods collected by Rudolf Szép. Karl Mergl, a professor at the lyceum in Bratislava, listed four species in his extensive manuscript focused on the flora and fauna of the city of Bratislava (Mergl 1940): *Porcellioscaber*, *P.laevis*, *Oniscusasellus* (as *O.murarius*) and *Armadillidiumvulgare* (as *Amadillovulgaris*).

The first synthesis of isopod fauna knowledge, including data from Slovakia, was conducted by Csiki (1926). He summarised data from the Hungarian Monarchy, reviewing samples from fifteen Slovak localities. During the first half of the 20^th^ century, faunistic data were collected by Dudich (1925, 1928, 1942, 1957), Méhelÿ (1929), Pongrácz (1936), and Kesselyák (1936). However, the most significant contributions to the taxonomy and biogeography of local isopod fauna were made by Verhoeff (1907, 1908, 1927a, b, 1937), Strouhal (1929a, b, 1939a, b, 1940a, b, 1947, 1948, 1951, 1953, 1964, 1965), and Frankenberger (1939, 1940, 1954, 1959, 1964). Strouhal (1940a, b) and Frankenberger (1940) provided the first comprehensive overviews of the Slovakian isopod fauna, briefly repeated by Babor (1943). Strouhal (1951) summarised woodlouse fauna knowledge in Central Europe and Frankenberger completed the work on Czech and Slovak fauna with a precise identification key (Frankenberger 1954) and monograph (Frankenberger 1959). Zdeněk Frankenberger was a decisive person defining modern faunistic and taxonomic research of terrestrial isopods in the former Czechoslovakia (Flasarová 1967).

Walter Černý, a prominent ornithologist and university teacher, also contributed to the study of terrestrial isopods. He provided collections to Frankenberger and reviewed his monograph (Frankenberger 1959). Černý’s unpublished collection, preserved in the National Museum in Prague, includes approximately 30 items (tubes) from Slovakia, containing 21 isopod species (nearly 2500 individuals) collected between 1931–1956 by various collectors. This material has been recently examined and the data were prepared for detailed publication (see Mock and Dolejš 2023).

After World War II, the study of isopods in Slovakia was continued by Ján Gulička, Miroslav Krumpál, and Marie Flasarová. Flasarová conducted faunistic and ecological studies (Flasarová 1980, 1986, 1994, 1998; Flasar and Flasarová 1989) and compiled an annotated bibliography and checklist of Slovak isopod fauna, published as an addition to her paper on the fauna of the Danube Lowland (Podunajská nížina) (Flasarová 1998). Her work includes short notes on findings in Slovakia, mainly focusing on the Czech fauna (Flasarová 1960, 1976, 1988, 1995). She was supported by influential Slovak zoologists Ján Gulička (Little Carpathians region), Ján Brtek (karst and lowlands of Slovakia) and Pavel Deván (Biele Karpaty Mts. and surroundings), who provided their collections. While Brtek and Deván apparently gave Flasarová their entire collections (Flasarová 1994), much of Gulička’s collection remains unprocessed in his estate, located in the Natural History Museums in Bratislava and Liptovský Mikuláš and the Department of Zoology, Faculty of Natural Sciences, Comenius University in Bratislava. Rudy et al. (2021) analysed these items, with the other material from isopods sampled in Slovak caves, stored at the affiliated laboratory of the first author of this study. A small collection of isopods from the Košel estate in Bratislava was analysed by Mock (2023).

In the 21^st^ century, terrestrial isopods in Slovakia have been frequently studied as part of cave arthropod assemblages. Comprehensive summaries of these studies have been provided by Košel (2007, 2009, 2012) and Kováč et al. (2014a). The unique invertebrate communities found in underground scree slopes have also been a source of valuable information on terrestrial isopods (Rendoš and Mock 2010, 2012; Rendoš 2012, 2016; Rendoš et al. 2016, 2019; Rudy et al. 2018). Tóth (2019) studied community isopods within the assemblage of macrofauna of solifluction volcanic debris.

The cave isopod *Mesoniscusgraniger* Frivaldszky, 1965, is particularly well studied in Slovakia. Extensive research covers its distribution, taxonomy, food preferences, gut content, cuticle anatomy, and life history, incorporating data from Slovak localities (Staněk 1932a, b; Kettner 1936; Balthasar and Frankenberger 1937; Arcangeli 1939; Gruner and Tabacaru 1963; Gulička 1975, 1978, 1982; Košel 1975, 1976, 1994, 2006; Gaál 1978, 1987, 2000; Hůrka and Pulpán 1978; Pomichal 1982; Mlejnek 1999, 2000, 2002; Gaál et al. 2000; Giurginca 2000; Franc and Mlejnek 2000; Mlejnek and Ducháč 2001, 2003; Elhottová et al. 2003, 2004; Giurginca 2005; Krištůfek et al. 2005; Nováková et al. 2005; Šustr et al. 2005, 2009; Balciar 2007; Piksa and Farkas 2007; Papáč 2008; Lukešová and Nováková 2009; Višnovská and Barlog 2009; Giurginca et al. 2012, 2015, 2016; Smrž et al. 2015; Tajovský and Mock 2015; Bella et al. 2016, 2022; Derbák et al. 2018; Ratkovský et al. 2019; Gaál et al. 2020). *Mesoniscusgraniger* and its associated species are also noted in biodiversity studies of caves (Košel 1984; Kováč et al. 2001, 2002a, b, 2004, 2005a, b, c, 2006, 2008, 2010b; Mock et al. 2002, 2004a, b, 2009; Lukáň et al. 2004; Papáč et al. 2006, 2007, 2009, 2014, 2019, 2020). Although *M.graniger* is absent in some regions with karst or non-karst underground habitats, common epigeic terrestrial isopods are sometimes found in shallow underground areas (Kováč et al. 2003, 2014b; Mock et al. 2003, 2005; Ľuptáčik et al. 2005; Papáč 2006, 2018; Papáč et al. 2015; Višňovská et al. 2017; Galabová 2022; Pribišová 2022).

The diversity and distribution of terrestrial isopods in Slovak caves were summarized by Rudy et al. (2021) and updated by Melega et al. (2022), incorporating new data from various collectors, including the estates of Gulička and Václav Ducháč. Studies on the phenology of *M.graniger* and co-occurring species, as well as their distribution along the depth gradient in limestone talus habitats, have been conducted by Mock et al. (2015), Rendoš et al. (2016) and Rudy et al. (2018).

Terrestrial isopods have also been found associated with the rhizosphere of plants in spring fens (Axamská 2021).

Woodlice assemblages have also been used to indicate environmental changes (Gulička 1957, 1960; Krumpál 1973, 1983; Čarnogurský et al. 1994; Majzlan and Hošták 1996; Jedlička et al. 1999; Jurík 2001; Snopková 2001; Stašiov 2001; Tajovský 2002; Majzlan et al. 2004; Dubovský 2005; Tuf and Tufová 2005; Holecová et al. 2005, 2012; Drinková 2006; Štrichelová and Tuf 2012; Martinka 2022; Martinka et al. 2022, 2023a, b). Haľková-Valkay et al. (2022) investigated isopods in leaf litter during winter.

Krumpál (1976, 1977) studied the reproductive biology and development of woodlice, proposing a method for evaluating biomass and length (Krumpál and Žitňanský 1977). Šustr et al. (2005) and Hámorský (2010) conducted eco-physiological experiments on some autochthonous isopod species.

Detailed faunistic studies have been conducted in specific regions, such as Tekov County (Comitat Bars) (Kesselyák 1936; Dudich 1957), the Malé Karpaty Mts. (Little Carpathians) (Mišík et al. 1974; Flasarová 1986; Flasar and Flasarová 1989; Kuracina and Kabátová 2005; Tuf and Tufová 2005; Majzlan et al. 2011; Štrichelová and Tuf 2012), the Nízke Beskydy Mts. (Krumpál 1975; Mock 2020), the Slovak Karst and Muránska Plateau (Gulička 1985), the Danube Lowland (Flasarová 1998; Gogová 2004), the Burda Mts. (Flasarová 1998; Mock 2017; Mock et al. 2020), the Pieniny Mts. (Hudáková and Mock 2006) and the Cerová vrchovina Highland (Tajovský and Mock 2015). Other notable studies include those on the isopod fauna of the Biele Karpaty Mts. (Tajovský et al. 2018), the Eastern Slovak Plain and Zemplínske vrchy Mts. (Mock et al. 2021), and the Čierna hora Mts. (Timková 2008; Timková and Mock 2008). Topp et al. (2006) studied isopod diversity in central Slovakia’s primeval forest. Turis and Vidlička (2013) noted *Protracheoniscuspolitus* in invertebrate assemblage associated with rare endemic plant, *Cyclamenfatrense*. Some species were mentioned in the general characteristics of protected areas (Čaputa 1987, 1988, 1991; Hudec 2002; Kováč et al. 2010a; Lantos et al. 2010) or Slovakia overall (Ferianc et al. 1972; Vilček and Hudec 2019). Mock et al. (2007) compared isopod biodiversity between faunistically well-studied regions in Poland and Slovakia.

Woodlice in synanthropic sites such as urban soils (Krumpál 1993), forest fragments in cities (Čemesová 2001; Jakabová 2001; Remešicová 2011; Štrichelová and Tuf 2012; Plutinská 2015), house interiors (Krumpál and Krištofík 1982, 1985; Krumpál et al. 1985), compost heaps (Rudy 2016; Rudy and Mock 2016) and greenhouses (Krumpál et al. 1997, 1999; Palkovičová and Mock 2008; Droběnová and Mock 2009) have also been noted. Additionally, studies on fauna in bird and mammal nests and burrows have included terrestrial isopods (Nosek and Lichard 1962; Fenďa et al. 1998; Cyprich et al. 2000; Vaníková 2009; Mock et al. 2008, 2021).

Historical data have been recently published, such as the collection of Czech arachnologist František Miller, revised by Dolejš and Tuf (2018), documenting twelve common species in northwest Slovakia and the Slovak Karst. Many other papers list terrestrial isopods faunistically or mention a few species marginally.

Data on terrestrial isopods of Slovakia have also been extracted from unpublished student theses defended at various universities, including Pavol Jozef Šafárik University in Košice, Comenius University in Bratislava, Technical University in Zvolen, Palacký University Olomouc, Technical University of Ostrava, and Prešov University in Prešov. All theses have been checked by the authors of this paper.

Secondary data on terrestrial isopods in Slovakia can also be found in monographs or papers focused on isopod fauna of other European regions (e.g., Wächtler 1937; Urbański 1950; Dominiak 1962; Gruner 1966; Schmölzer 1965; Radu 1983, 1985; Flasarová 1995; Tomescu et al. 2015, 2016 and Giurginca 2022), in the worldwide catalogue of terrestrial isopods (Schmalfuss 2003) or in the determination key of invertebrates from the Czech and Slovak republics (Buchar et al. 1995). Štrichelová (2010) tried to outline the characteristics of the isopodofauna of the Western Carpathians, while Kseňáková (2011) collected published data on the occurrence of representatives of the Trichoniscidae family in Slovakia.

Since 2000, a small informal working group of isopodologists from the Czech and Slovak republics has been meeting together with myriapodologists with a frequency of approximately one and a half years at workshops that include presentations with abstract proceedings, discussions, and field mapping alternately at locations in the Czech Republic and Slovakia. The last, thirteenth, meeting of the isopologic community took place in Hostomice, Czech Republic in 2003 (for proceedings and history of meetings see Dolejš 2023).

This study presents a critical list of the fauna with an analysis of habitat preferences, bioindication potential and biogeography, with notes on the occurrence of rare species, dubious literature data and first recorded species.

## ﻿Methods

We thoroughly reviewed all taxonomic, faunistic and ecological literature from 1794 to the end of 2023 to compile a comprehensive bibliography of terrestrial isopods identified to the species level within present-day Slovakia. This bibliography includes more than 200 publications and more than 20 unpublished university theses. We excluded abstracts from scientific congresses and meetings if their topics were later published in regular articles. The resulting checklist was critically reviewed based on current taxonomy, ecology, and distribution knowledge, as well as field experiences. The nomenclature aligns with the views of authorities like Schmalfuss (2003) and Boyko et al. (2024).

Collections of terrestrial isopods from Slovakia are housed in various museums:

- The Natural History Museum in London, United Kingdom
- The Hungarian National History Museum in Budapest, Hungary
- The National Museum in Prague, Czech Republic
- The Natural History Museum in Vienna, Austria
- The East Slovak Museum in Košice, Slovakia
- The Slovak Museum of Nature Protection and Speleology in Liptovský Mikuláš, Slovakia

The most extensive unpublished collection is in the National Museum in Prague, primarily due to the estates of Z. Frankenberger, W. Černý, and M. Flasarová. Additionally, significant collections are maintained in the authors’ institutions.

## ﻿Results and discussion

**Figure 1.**
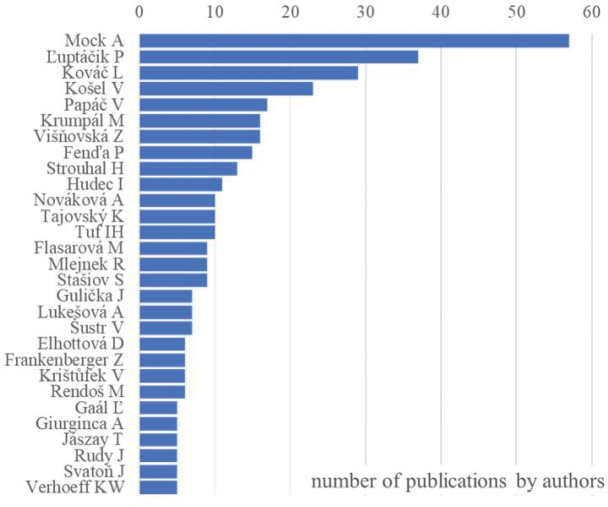
List of the most active authors publishing five or more publications on Slovak terrestrial isopods (1794–2023).

**Figure 2.**
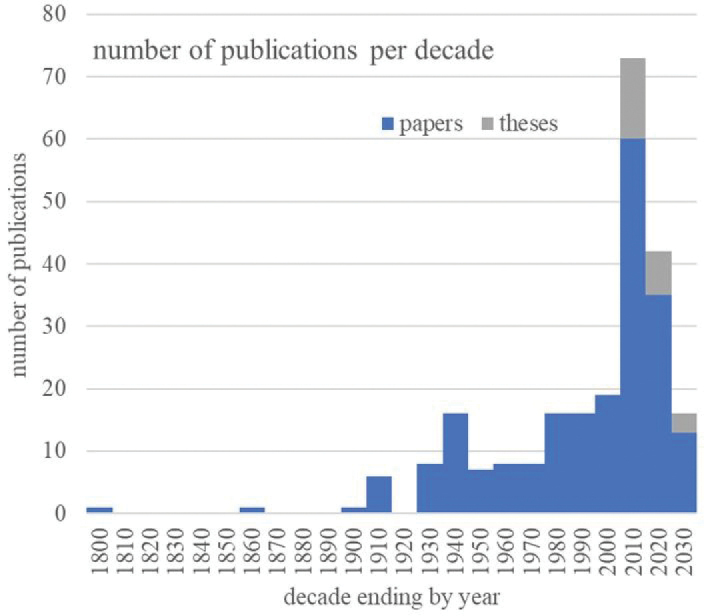
Development of interest about Slovak terrestrial isopod fauna represented by the number of papers published. Counts of papers are summarised in decades, complete list contains 221 papers and 25 theses.

We found 213 publications documenting terrestrial isopod species in Slovakia, authored by 191 individuals. Of these, 127 authors contributed to just one publication each, while 29 authors had five or more publications (Fig. 1). Notably, five of the most prolific authors were speleologists who extensively sampled cave fauna throughout Slovakia. The interest in Slovakian terrestrial isopods peaked at three distinct times (Fig. 2): around the turn of the 19^th^ and 20^th^ centuries, just before World War II and at the start of the 21^st^ century. The most recent peak reflects a surge in speleological research and university-driven studies, with 25 theses defended primarily at the universities in Košice, Bratislava (Slovakia), and Olomouc (Czech Republic).

Summarizing all published and unpublished data, we compiled a list of 48 woodlice species from 14 families in Slovakia (Table 1). When comparing woodlouse fauna of neighbouring countries, species counts vary: 57 in Hungary (Forró and Farkas 1998; Farkas and Vilisics 2013), 43 in the Czech Republic (Orsavová and Tuf 2018), 34 in Poland (Jażdżewski and Konopacka 1993) and 96 in Romania (Giurginca 2022). These differences are influenced by several factors, including the extent of faunistic knowledge, country dimensions and heterogeneity, landscape naturalness, latitude position and proximity to main biorefugia like the Balkan Peninsula the Alps and the Carpathians. The presence of introduced species correlates with the urbanization of the environment and trade traffic (Sfenthourakis and Hornung 2018).

**Table 1.** List of terrestrial isopods (Oniscidea) documented in Slovakia with geographical and ecological characteristics.

Families	List of species	Regional chorotypes	Global chorotypes	Relictness	Habitat
**Ligidiidae**	*Ligidiumgermanicum* Verhoeff 1901	M	C+SE Europe	R	forest (leaf litter)
	*Ligidiumhypnorum* (Cuvier, 1792)	M	W Palearctic	A	forest (leaf litter)
	*Ligidiumintermedium* Radu, 1950	M	**Carpathian**	R	forest (leaf litter)
**Mesoniscidae**	*Mesoniscusgraniger* (Frivaldszky, 1865)	S	**Carpathian +Dinaric**	R	cave, scree
**Styloniscidae**	*Cordioniscusstebbingi (Patience, 1907)	U	SW Europe	E	greenhouse
**Trichoniscidae**	*Androniscusroseus* (C. Koch, 1838)	U	S Europe	E	rotten wood
	*Haplophthalmusdanicus* Budde-Lund, 1880	U/L	Europe	E	rotten wood
	*Haplophthalmusmengii* (Zaddach, 1844)	M	W Palearctic	A	forest (rotten wood)
	*Hyloniscusmariae* Verhoeff, 1908	M	**Carpathian**	R	forest (leaf litter)
	*Hyloniscusriparius* (C. Koch, 1838)	L/M/U	C+E Europe	E	forest (leaf litter)
	*Hyloniscustranssilvanicus* (Verhoeff, 1901)	L/M	**Carpathian**	R	forest (leaf litter)
	*Miktoniscuslinearis (Patience, 1908)	U	unknown	E	greenhouse
	*Trichoniscoidesalbidus (Budde-Lund, 1880)	U	W Europe	E	greenhouse
	*Trichoniscuscarpaticus* Tabacaru, 1974	M/L	**Carpathian**	R	forest (leaf litter)
	*Trichoniscusnoricus* Verhoeff, 1917	U	C+S Europe	R	forest (leaf litter)
	*Trichoniscusprovisiorus* Racovitza, 1908	U	W Palearctic	R	forest (leaf litter)
	*Trichoniscuspusillus* Brandt, 1833	U/L/M	E Europe	E	forest (leaf litter)
	*Trichoniscuspygmaeus* Sars, 1898	S	W Palearctic	R	cave. soil
**Buddelundiellidae**	^1^ *Buddelundiellacataractae Verhoeff, 1930	U	S Europe	E	greenhouse
**Oniscidae**	*Oniscusasellus* Linnaeus, 1758	U	N+W Europe	A	rock
**PHILOSCIIDAE**	*Lepidoniscusminutus* (C. Koch, 1838)	M	C+S Europe	R	forest (leaf litter)
**Platyarthridae**	*Platyarthrushoffmannseggii* Brandt, 1833	U/L	W Palearctic	E	grassland
	*Trichorhinatomentosa (Budde-Lund, 1893)	U	Tropical America	E	greenhouse
**Porcellionidae**	*Porcelliodilatatus* Brandt, 1833	U	S Europe	E	grassland, rock
	*Porcelliolaevis* Latreille, 1804	U	Mediterranean	E	grassland. shrub
	^2^*Porcelliomontanus* Budde-Lund, 1885	M	Alp’s surroundings	R	forest (leaf litter)
	*Porcellioscaber* Latreille, 1804	U	SW Europe	E	rock (walls)
	*Porcelliospinicornis* Say, 1818	U	Europe	E	rock (walls)
	*Porcellionidespruinosus* (Brandt, 1833)	U/L	S Europe	E	grasslands
**ARMADILLIDIIDAE**	*Armadillidiumnasatum Budde-Lund, 1885	U	S Europe	E	grassland
	*Armadillidiumopacum* (C. Koch, 1841)	M	Europe	R	forest (leaf litter)
	^2^*Armadillidiumpictum* Brandt, 1833	M	C+W Europe	R	rocky grassland
	*Armadillidiumversicolor* Stein, 1859	U/L/M	C+S Europe	E	rock
	*Armadillidiumvulgare* (Latreille, 1804)	U/L/M	S Europe	E	rock
	*Armadillidiumzenckeri* Brandt, 1833	L	E+C Europe	R	marshland
**Armadillidae**	^1^ *Reductoniscuscostulatus Kesselyák, 1930	U	Paleotropical	E	greenhouse
**Trachelipodidae**	*Naguruscristatus (Dollfus, 1889)	U	Pantropical	E	greenhouse
	*Porcelliumcollicola* (Verhoeff, 1907)	L/M	C+SE Europe	E	grassland, shrubs
	*Porcelliumconspersum* (C. Koch, 1841)	L/M	C Europe	A	forest (leaf litter)
	^2^*Trachelipusarcuatus* (Budde-Lund, 1885)	M?	Mediterranean	R	forest (leaf litter)
	*Trachelipusdifficilis* (Radu, 1950)	M	**Carpathian**	R	rock
	*Trachelipusnodulosus* (C. Koch, 1838)	L/M	C+S Europe	A	grassland
	*Trachelipusrathkii* (Brandt, 1833)	U/L/M	Europe excl. Mediterranean	E	forest (leaf litter)
	*Trachelipusratzeburgii* (Brandt, 1833)	M/L	C+E Europe	R	forest (litter, barks)
**Agnaridae**	*Orthometoponplanum* (Budde-Lund, 1885)	L/M	NW Mediterranean + Pannonian	R	shrubs, rocks
	*Protracheoniscusmajor (Dollfus, 1903)	U	C+W Asia	E	rock (walls)
	*Protracheoniscuspolitus* (C. Koch, 1841)	M	C+S Europe	R	forest (leaf litter)
**Cylisticidae**	*Cylisticusconvexus* (De Geer, 1778)	U/L/M/S	W Palearctic	E	rock (walls)

**Notes**: ^1^ the first record, ^2^ revision of the occurrence needed, *exotic species (originated in different zoogeographic area and/or in the other climate zone). **Regional chorotypes** (following definition by Fattorini (2015)) distinguished species occurring in Slovakia into four groups, three with natural proposal distribution, subterranean (S), lowland landscape as cultural steppe, fragment of woods and marshlands (L), and mountainous landscape with zonation of vegetation and predominancy of woodland (M) and urbanized environment, created or strongly influenced by human communities (built-up areas, urban greenery, brown field) (U). Urban environment and, to a lesser extent, lowland environments are the open doors for non-native (alien) species to establish. **Global chorotypes** (species distribution area) refer the natural area of species mainly according to Schmalfuss (2003) and Forró and Farkas (1998). **Relictness** means the rate of nature of the occurrence and relation the habitat type (see Tuf and Tufová 2008): R = relic species (exclusive dweller of natural sites), A = adaptable species (preference of natural sites, colonising connected sites moderately arranged by humans), E = eurytopic, opportunistic species (able to colonise or even prefer various sites including these strongly modified by humans).

Mountainous areas in Slovakia are particularly diverse in isopod species, hosting 24 species, half of which are exclusive to these regions and likely autochthonous. In contrast, lowland areas are less diverse, with 16 species, among which *Armadillidiumzenckeri* is native and exclusive to these areas, where it prefers wetlands. Lowlands are more affected by human activities, resulting in higher proportions of both eurytopic and non-native species. Urban environments support a significant number of isopod species, with a notable presence of alien, thermophilic, and xerotolerant species.

The natural subterranean environment has lower species diversity. Central region caves and screes host the cavernicolous species *Mesoniscusgraniger* (Rudy et al. 2018, 2021) and an unidentified blind subterranean species from the family Trichoniscidae (Košel et al. 2007; Melega et al. 2022), although the latter is not included in Table 1 due to the absence of the male specimens needed for proper identification.

### ﻿New records

**Figure 3.**
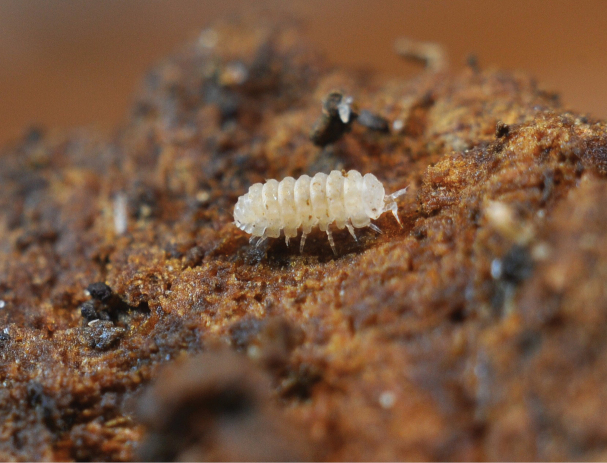
*Buddelundiellacataractae*, Botanical Garden of the Pavol Jozef Šafárik University in Košice (photograph Ľ. Kováč and A. Mock).

**Figure 4.**
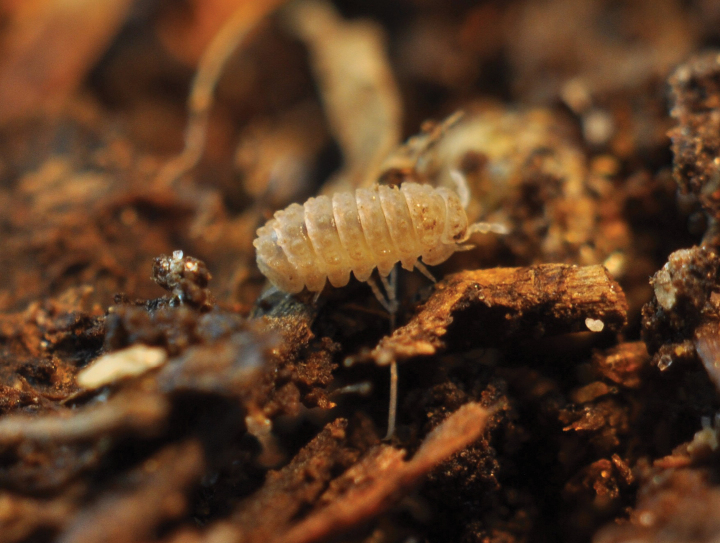
*Reductoniscuscostulatus*, Botanical Garden of the Pavol Jozef Šafárik University in Košice (photograph Ľ. Kováč and A. Mock).

Two new terrestrial isopod species were documented in Slovakia for the first time, collected during a visit to the Pavol Jozef Šafárik Botanic Garden in Košice on 7 February 2017. Numerous females and juveniles of both species were found in a greenhouse with tropical vegetation, in humus soil and under rotten wood. These species are *Buddelundiellacataractae* Verhoeff, 1930 (Fig. 3; autochthonous in the Mediterranean region) and *Reductoniscuscostulatus* Kesselyák, 1930 (Fig. 4; originally from tropical regions of the Oriental zone), both introduced in other parts of Europe, especially under the Atlantic climate. Both species are tiny (2–3 mm), pale, hemispherical and roll into a sphere when disturbed. Their introduction is likely recent, as they were not recorded in previous inspections (Droběnová and Mock 2009). Subsequent inspections confirmed their establishment in the greenhouse.

### ﻿Taxa described from Slovakia

Several taxa have been described from Slovakia, primarily by K. W. Verhoeff and H. Strouhal. Many of these are now considered junior synonyms (see Schmalfuss 2003):

- ***Ligidiumcarpathicum* Verhoeff, 1937**: Type locality: Strečno, Váh River Valley. Junior synonym of *Ligidiumhypnorum* (Cuvier, 1792) (Bonnefoy and Marchal 1943).
- ***Haplophthalmusverhoeffi* Strouhal, 1948**: Type locality: Kováčov, SW Slovakia. Junior synonym of *Haplophthalmusmengii* (Zaddach, 1844). Legrand et al. (1950) compared the type specimens of *H.verhoeffi* with *Haplophthalmusperezi* Legrand, 1943, finding slight morphological differences. They observed the similarity but not the identity of both taxa. Recently, both names have been considered junior synonyms of *Haplophthalmusmengii* (Zaddach, 1844).
- ***Hyloniscusmariae* Verhoeff, 1908**: Type locality: Belianske Tatry Mts. Accepted species. The syntype (male) is deposited in the Natural History Museum in London (Britain) as a part of Verhoeff’s collection of isopods (Ellis and Lincoln 1975).
- ***Protracheoniscussaxonicusslovakius* Strouhal, 1940**: Type locality: Krompachy. Accepted as *Protracheoniscuspolitusslovakius* Strouhal, 1940.
- ***Lepidoniscuscarpathicus* Strouhal, 1940**: Type locality: Krompachy. Junior synonym of *Lepidoniscusminutus* (C. Koch, 1838).
- ***Lepidoniscusgermanicusroubali* Strouhal, 1940**: Type locality: middle section of the Hron River, Central Slovakia. Taxonomic status uncertain (Boyko et al. 2024).

### ﻿Dubious and questionable data

Some species in Table 1 are marked as questionable due to their location outside a continuous species range, lack of repeated findings, or single-specimen documentation. Notable examples include (see Frankenberger 1959):

- ***Porcelliomontanus*** : One male from the town Žilina, possibly mislocated due to confusion with the village Žilina in the Czech Republic.
- ***Trachelipusarcuatus*** : A questionable find in the Slovak Karst, with incomplete specimens making identification uncertain.

### ﻿Expected species

Additional woodlice species are expected in Slovakia, particularly in the family Trichoniscidae. Unidentified juveniles from the genera *Androniscus* and *Trichoniscus* suggest the presence of potentially new species. The genus *Haplophthalmus* needs revision due to the complexity of the *H.mengii* species complex. For example, *H.hungaricus* Kesselyák, 1930, was recognised in the material collected near the southern border of Slovakia in Hungary (Vilisics et al. 2008). The myrmecophilous species *Platyarthrusschoblii* Budde-Lund, 1885 may also occur, spreading with the garden ant *Lasiusneglectus* Van Loon, Boomsma & Andrasfalvy, 1990 (Hornung et al. 2005).

### ﻿Zoogeographic notes

Most woodlice species in Slovakia have a Central European to European distribution, with some species considered Carpathian endemics. The Carpathians’ status as a biodiversity hotspot is noted, but endemic species are fewer compared to regions like the Romanian Carpathians. Future research, including molecular taxonomy, is needed to clarify the true extent of endemism and species diversity.

### ﻿Ecological classification

Slovak isopod fauna is classified into relict, adaptable and eurytopic species (Table 1). Eurytopic species dominate (24 species), followed by relict (19 species) and adaptable (5 species). Native relict species predominantly inhabit montane forests (Hudáková and Mock 2016), wetlands (Mock et al. 2021) and natural subterranean habitats (Rudy et al. 2018, 2021). Urban and transformed landscapes host mainly adaptable and eurytopic species, often non-native (Kseňáková 2014). Poor isopod fauna characterizes agrocenoses (Flasarová 1998; Mock et al. 2021), coniferous forests, and high-altitude Carpathian habitats (Mock 2008; Timková 2008; Válentová 2013). Relict species are vulnerable and are included in the forthcoming Red Book of Slovak Invertebrates (Mock et al. 2023; Kokavec et al. 2024).

## ﻿Conclusions

The current study comprehensively analyses the bibliography of terrestrial isopods in Slovakia, culminating in a list of 48 species, including two species documented in Slovakia for the first time based on the authors’ collections. The checklist is critically reviewed and updated in accordance with current taxonomic standards and field experience. The study highlights species with dubious records due to inconsistencies in location data or identification, emphasising the need for further verification through precise field research. This exhaustive bibliography and species list of terrestrial isopods in Slovakia holds significant potential for enhancing both taxonomic clarity and ecological insights, thereby aiding in the conservation and study of Slovakia’s natural habitats.

## ﻿Additional information

### Author ORCIDs

Andrej Mock https://orcid.org/0000-0002-6541-9633

Karel Tajovský https://orcid.org/0000-0002-3619-3815

Adrián Purkart https://orcid.org/0000-0002-8471-5083

Ivan Hadrián Tuf https://orcid.org/0000-0003-0250-0482

### Data availability

All of the data that support the findings of this study are available in the main text.
